# The Doctor’s Side of It

**Published:** 1917-04

**Authors:** C. H. Mackintosh


### The Doctor's Side Of It.

Laugh if you like at the doctor's mistakes—
And I reckon we all make a few—
He's giving the universe more than he takes,
Which is more than the most of us do!

Feather your arrows with humorous chaff
And tip them with satire and bile,
But don't ask your target to join in the laugh
He's entirely too busy to smile!

For General Practitioner, Army of Health,
Is fighting the terrors you fear.
While you are discussing his "ill gotten wealth"
(Most likely a thousand a year!)

He's saving you sickness and giving you strength
And it's easy to laugh when you're strong;
But one of your terrors may get you at length
And after the pitch of your song!

When you will remember the jests you have made
And scorn his assistance, no doubt!
Or you will entreat him to fly to your aid
With the skill you have jested about?

—*C. H. Mackintosh in Judge.*

---

Those who insist that the South is always behind the North should visit the Austin Presbyterian Sanitarium and inspect its up-to-date equipment in the operating rooms and become convinced that it is keeping well up with the North in its service to physicians and patients.

The Sanitarium has recently installed a big Gwathmey Gas-Oxygen apparatus, which is said to be one of the most perfect machines of its kind on the market today. It is being largely used in the East and North by the leading anesthetists.

The physicians in Austin, and the physicians in the surrounding towns, who do work in the Sanitarium will be glad to know that they can give their patients the advantage of gas-oxygen anesthesia without the element of danger heretofore attendant upon its use.

We congratulate the Sanitarium and the physicians who do their operating there upon the installation of this new apparatus.

